# Management of Large Erupting Complex Odontoma in Maxilla

**DOI:** 10.1155/2014/963962

**Published:** 2014-12-14

**Authors:** Colm Murphy, John Edward O'Connell, Edward Cotter, Gerard Kearns

**Affiliations:** ^1^Department of Oral and Maxillofacial Surgery, Our Lady's Children's Hospital, Crumlin, Dublin 12, Ireland; ^2^Hermitage Medical Clinic, Suite 10, Old Lucan Road, Dublin 20, Ireland

## Abstract

We present the unusual case of a large complex odontoma erupting in the maxilla. Odontomas are benign developmental tumours of odontogenic origin. They are characterized by slow growth and nonaggressive behaviour. Complex odontomas, which erupt, are rare. They are usually asymptomatic and are identified on routine radiograph but may present with erosion into the oral cavity with subsequent cellulitis and facial asymmetry. This present paper describes the presentation and management of an erupting complex odontoma, occupying the maxillary sinus with extension to the infraorbital rim. We also discuss various surgical approaches used to access this anatomic area.

## 1. Introduction

Odontomas are benign tumours of odontogenic origin [1–3]. They are described as mixed tumours containing both epithelial and mesenchymal elements [3]. These calcified lesions are classified into compound and complex types. Compound odontomas may appear as tooth like structures containing enamel, dentine, cementum, and pulp and may be single or multiple [4]. Complex odontomas appear as a disorganized amorphous mass of calcified hard tissues [4, 5]. Both lesions usually present in the first two decades of life [4]. Clinical presentation may be as an impacted tooth, alveolar swelling, or incidental radiographic finding. Complex odontomas tend to occur in the posterior mandible or maxilla and only rarely reach a considerable size [6]. Radiographically, the complex odontoma appears as a dense amorphous irregular mass with well-demarcated borders [6].

The differential diagnosis for mixed odontogenic tumours includes compound and complex odontoma, ameloblastic fibroma, and ameloblastic fibroodontoma [6, 7]. Compound odontomas are recognizable as orderly tooth like structures. A complex odontoma may bear resemblance to osteoblastoma, ossifying fibroma, and osteomata [6]. Ameloblastic fibroodontoma presents as a mixed radiolucent-radiopaque lesion and may resemble a developing odontoma. Ameloblastic fibroma is a separate entity and presents as a radiolucent lesion [6, 7].

Complex odontomas present less frequently than compound odontomas. In addition, eruption through the mucosa is rare [7]. To our knowledge, there have only been 20 reported cases of erupting odontoma in the literature, of which 11 were complex odontomas [8].

## 2. Case Report

**Figure 1:**
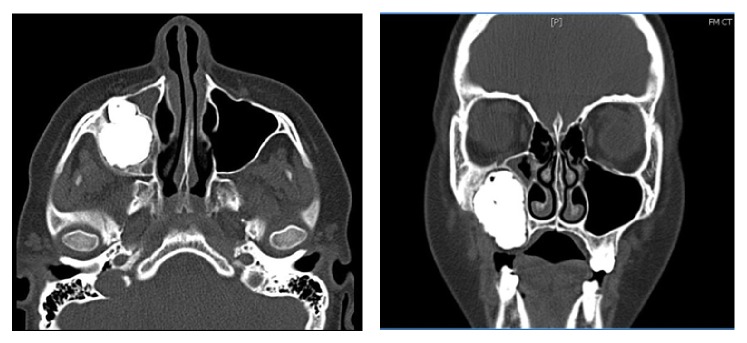
Axial and coronal CT images demonstrating the extent of the lesion in the right maxillary antrum.

**Figure 2:**
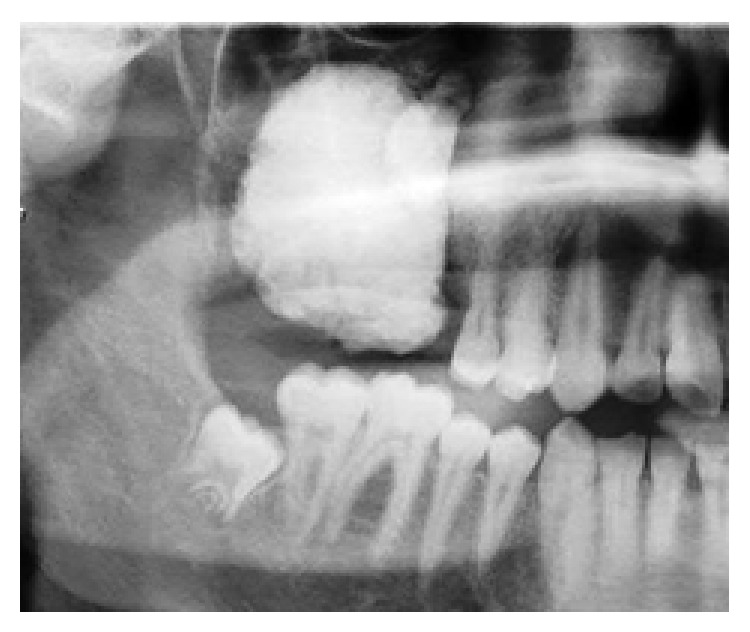
Orthopantomogram.

A 13-year-old boy was referred to the department of oral and maxillofacial surgery in a tertiary referral centre and presented with a right facial cellulitis, and a hard mass was palpable in his right maxilla The patient reported that the symptoms had begun 1 week previously but that he had noticed a mass in the right maxillary molar area 6 months previously. The infection was treated with empirical intravenous antibiotic therapy. Extraoral examination revealed a hard swelling over the right maxilla, with an associated intraoral calcified mass and missing molar teeth. There was no associated sensory nerve deficit. Plain radiographs and computed tomography demonstrated an extensive calcified lesion in the right maxilla, extending to the infraorbital rim (Figures 1 and 2). An incisional biopsy was taken and histopathological examination suggested the presence of odontoma or fibrous dysplasia.

**Figure 3:**
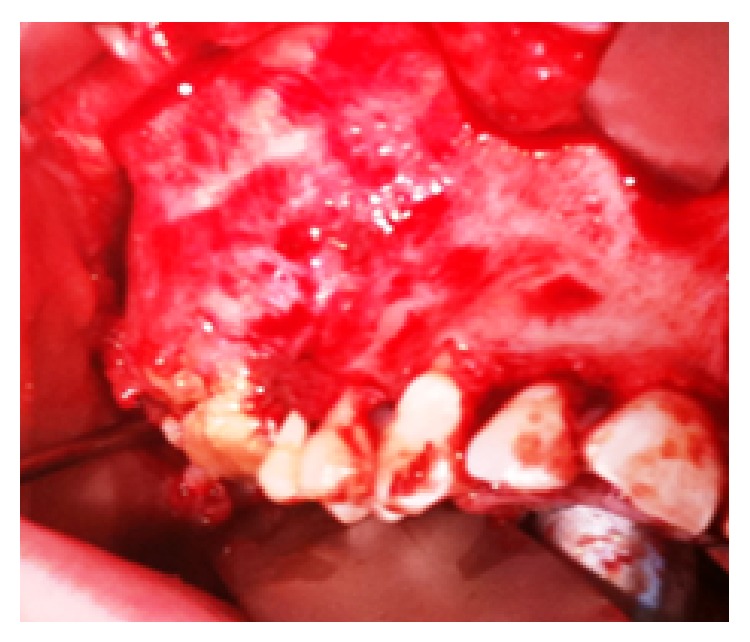
Intraoperative photo of lesion.

Surgical removal of the lesion was planned using a transoral approach under general anaesthesia. A mucoperiosteal flap was raised and the tumour was identified (Figure 3). The lesion was found to be eroding into the infraorbital rim. An upper lip split incision was made to facilitate complete removal of the tumour and maintain the integrity of the orbital floor and allow safe dissection and release of the infraorbital nerve. The tumour was enucleated intact using an osteotome and a periosteal elevator, while maintaining continuity of the infraorbital rim and orbital floor. The ipsilateral buccal fat pad was mobilised and advanced to repair the maxillary defect, following tumour removal.

**Figure 4:**
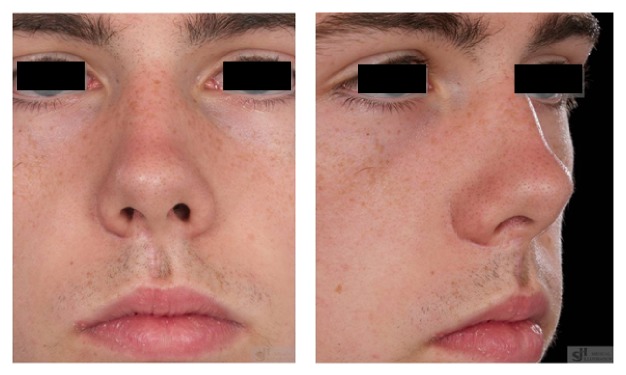
Postoperative photo demonstrating satisfactory appearance of extraoral incision.

Histopathological examination confirmed the diagnosis of complex odontoma. The patient made an uneventful recovery and had no sensory nerve deficit. The extraoral incision has healed well with minimal scarring; the patient is pleased with the appearance and healing of his extraoral incision (Figure 4). Prosthetic impressions were taken to facilitate construction of a maxillary obturator. To date, the patient has been followed up for a period of 36 months. There has been no clinical or radiographic evidence of recurrence. He will be considered for autogenous bone grafting and placement of osseointegrated dental implants and an overlying fixed prosthesis in the future.

## 3. Discussion

Odontomas are the most common odontogenic tumour [1–3]. They are characterized by slow growth and nonaggressive behaviour. They usually present in children and young adults in the 2nd decade of life [9, 10]. Complex odontomas are commonly found in the maxillary sinus and posterior mandible and can grow to a large size but rarely cause jaw deformity [1]. The treatment for odontomas is enucleation. Recurrences are rare.

The mechanism of eruption of odontoma is different to tooth eruption as Osteomata lack root formation and a periodontal ligament [4]. The increasing size may lead to resorption of the edentulous part of the alveolar process with subsequent exposure in the oral cavity [7]. This is the most likely explanation in our case due to the extensive nature of the lesion. Therefore, it could be suggested that, rather than erupting, the tumour simply erodes the adjacent bone leading to exposure in the oral cavity.

The surgical removal of benign tumours from the maxillary sinus has traditionally been carried out via a transoral approach with Caldwell-Luc antrostomy through the lateral sinus wall. However, large tumours, which occupy the entire antrum or cause significant deformity, may require a more extensive approach to facilitate surgical access. Korpi et al. [2] have advocated the use of the Le Fort 1 downfracture to gain access to the posterior maxilla for removal of complex odontoma. They suggest that this approach decreases the risk of bony defects, thus preventing oroantral fistula formation, and reduces facial deformity [2]. The maxilla can be repositioned in its original position with titanium miniplates and screws. However, we feel that downfracture of the maxilla is less predictable when there has been significant bone resorption and expansion due to a large tumour as it was in this present case.

The most common approach for management of maxillary odontogenic neoplasms is a transoral approach [7]. Labial and palatal mucoperiosteal flaps may be raised to allow adequate exposure. A transcutaneous approach may be required to facilitate the safe and adequate removal of more extensive maxillary odontogenic tumours. Extensions to the orbital floor and beyond the posterior wall of the maxillary sinus are examples [6].

In this present case, an upper lip split was performed to both adequately expose the tumour and maintain the integrity of the orbital floor, as well as allowing safe dissection and release of the infraorbital nerve. The patient suffered no sensory nerve deficit and the extraoral skin incision healed satisfactorily.

## 4. Conclusion

This case highlights the extensive nature and rare presentation of erupting complex odontomas. They may increase in size after calcification and lead to complications following eruption [4]. They may present with facial cellulitis or, more rarely, facial deformity. Surgical removal is the treatment of choice with preservation of adjacent structures. In cases with larger tumours, an extraoral skin incision may be successfully used to allow access to and removal of the lesion.
